# Fluids and Barriers of the CNS: a new journal encompassing Cerebrospinal Fluid Research

**DOI:** 10.1186/2045-8118-8-1

**Published:** 2011-01-18

**Authors:** Hazel C Jones, Tetsuya Terasaki

**Affiliations:** 1 Gagle Brook House, Chesterton, Bicester, OX26 1UF, UK; 2 Department of Biochemical Pharmacology and Therapeutics, Graduate School of Pharmaceutical Sciences, Tohoku University, Aoba 6-3, Aramaki, Aoba-ku, Sendai 980-8578, Japan

## Abstract

This article celebrates the re-launch of *Cerebrospinal Fluid Research *in its new format as *Fluids and Barriers of the CNS*. Editors-in Chief, Hazel Jones and Tetsuya Terasaki, anticipate that this expanded journal will provide a unique and specialist platform for the publication of research in cerebrospinal fluid and all brain barriers and fluid systems in both health and disease.

## Editorial

Welcome to *Fluids and Barriers of the CNS*, formerly *Cerebrospinal Fluid Research*!

### Achievements of Cerebrospinal Fluid Research 2004-2010

This month sees the re-launch of *Cerebrospinal Fluid Research *in its new format *Fluids and Barriers of the CNS*. Since its inception in December 2004 [1], *Cerebrospinal Fluid Research *has published 106 research, reviews, and commentary articles, and also supplements containing the proceedings of the Society for Research into Hydrocephalus and Spina Bifida. The journal has published manuscripts on cerebrospinal fluid (CSF) and choroid plexus in health and disease interpreted in the widest sense. This has included articles on CSF physiology, secretion, flow and absorption, fluid dynamics, and fluid composition both in health and in disease states such as hydrocephalus, neurodegenerative diseases and CNS infections. CNS disorders that impact the CSF system such as neural tube defects, Chiari malformation and syringomyelia have also been included. Articles on the choroid plexus, either *in vivo *or *in vitro *have included topics such as junctional proteins, transport systems, gene expression, receptors and peptide secretion. All articles are open access; i.e. freely available to anyone with access to the internet, and previously published articles are fully indexed and will be accessible from the new journal platform.

### Journal expansion into Fluids and Barriers of the CNS

After discussions with the current editorial board and the journal's publisher, BioMed Central (part of Springer Science+Business Media), we have decided to expand the journal's scope into all brain barriers and to re-launch with a new title, *Fluids and Barriers of the CNS (FBCNS) *http://www.fluidsbarrierscns.com. Homeostatic maintenance of the internal environment of the nervous system through normal functioning of brain fluids and barriers is vital for health of the organism. Hitherto, the subject area of brain fluids and brain barriers has not had a specialist platform. Several thousands of articles on these topics are published every year in a variety of different journals; we hope to bring a good proportion of these under one umbrella into *FBCNS*. We recognise that the scientific community working in the research fields of the blood-brain barrier, the blood-retinal barrier, the blood-nerve barrier together with the blood-cerebrospinal fluid barrier and the role of the cerebrospinal fluid and the brain interstitial fluid, is currently not especially large. However, we expect significant synergistic effects among those scientists with this new publishing format, by facilitating the progress of the research and simultaneously providing more opportunities to expand the community to those with fringe interests. Accordingly, our aim is to bring the field under one comprehensive umbrella offering quality peer-reviewed manuscripts on all aspects of fluids and barriers of the CNS.

### Scope for FBCNS

*FBCNS *will continue to publish articles on all aspects of CSF and choroid plexus in health and disease. The cerebrospinal fluid, its composition, circulation and absorption, has multiple roles in both normal and abnormal brain function and is closely associated with the neuronal interstitial fluid. From now *FBCNS *is, in addition, **actively seeking manuscripts on all CNS barrier systems **including the blood-CSF barrier, the blood-brain barrier, the inner and outer blood-retinal barriers, and the blood-nerve barrier which perform functions such as fluid secretion, chemical signalling, physical and chemical buffering, specialised directional transport and facilitation of immune surveillance. Particularly interesting are topics such as how the barrier tight junctions are constructed and regulated in pathophysiological conditions and how different cells and cell types interact to maintain a dynamic barrier function (cell-cell direct interaction and paracrine effects). This includes biochemical and physiological studies on the interactive mechanisms between endothelial cells, astrocytes and pericytes at the blood-brain barrier, and between endothelial cells, Müller cells and pericytes at the inner blood-retinal barrier. It is important to explore the similarities and differences between the blood-CSF barrier, i.e., choroid plexus epithelial cells, and the outer blood-retinal barrier, retinal pigment epithelial cells and also the similarities and differences between the blood-brain barrier, and the inner blood-retinal barrier, and the blood-nerve barrier. The fluids and barrier systems are important in neurodevelopment and neurodevelopmental disorders such as hydrocephalus and neural tube defects, in aging and aging disorders such dementia, Alzheimer's, Parkinson's disease and normal pressure hydrocephalus. Throughout all stages of life they play an important role in brain inflammation, brain injury and repair, and neurodegenerative diseases such as multiple sclerosis. Manipulation of the blood-brain barrier can provide a route for drug delivery to the brain. Chemical analysis of the CSF can potentially be used for the diagnosis of neurological diseases, and may provide a supplementary route for drug delivery to the CNS.

### New Co-Editor in Chief and restructured editorial board

To facilitate this expansion, Tetsuya Terasaki, Distinguished Professor, Tohoku University, Japan, has recently joined the journal as joint-Editor-in-Chief. Dr. Terasaki has published a number of articles on transporter function and tight junction proteins in the blood-brain barrier and the blood-cerebrospinal fluid barrier. He has published as a co-author, studies on the blood-retinal barrier and the blood-nerve barrier which have been performed by his colleagues. He also recognises the importance of technological advances and development to facilitate barrier research. He will have responsibility for the publication of articles on neural barriers including the blood-brain, blood-nerve and blood-eye barriers. Furthermore, we are delighted to have Distinguished Professor William M. Pardridge (UCLA School of Medicine, USA), Professor Joan Abbott (King's College London, UK), Professor Yuichi Sugiyama (University of Tokyo, Japan), world-leaders of these research fields, who will act as Honorary Advisors to the journal by giving advice on policy, promoting submissions and acting as special advocates. Along with these, we are now recruiting people with BBB expertise to our international Editorial Board http://www.fluidsbarrierscns.com/about/edboard and aim to have a broad range of expertise in all relevant fields.

### Publishing in Fluids and Barriers of the CNS

We strongly encourage you to submit any articles relating to the scope of our journal, *Fluids and Barriers of the CNS *including research articles, review articles, commentaries, book reviews and meeting proceedings. Each manuscript will be reviewed by a member of the Editorial Board or, where appropriate, allocated to external reviewers. Usually two or three reviewers, who remain anonymous, will be sought for each manuscript and authors are asked to provide a list of suggested reviewers on submission of their manuscript. All articles are published immediately upon acceptance. Articles are archived in PubMed and the US National Library of Medicine's full-text repository of life science literature. The journal is also participating in the British Library's e-journals pilot project, and plans to deposit copies of all articles with the British Library. Publications are tracked by PubMed Central, CAS, EMBASE, Scopus and Google Scholar. BioMed Central is working closely with Thomson Reuters (ISI) to ensure that citation analysis of articles published in *FBCNS *will be available.

### About open access

Open Access defines the way in which articles are published. All articles become freely and universally accessible online, and an author's work can be read by anyone at no cost. Second, authors hold the copyright for their work and can grant anyone the right to reproduce and disseminate the article, provided that it is correctly cited and no errors are introduced [2]. Because no subscription is needed to access articles, article processing charges (APCs) are charged to authors or their institutions to cover the costs of publication. The price charged is very competitive and among the lowest charged by other publishers offering open access. Waivers can be given under special circumstances and by special request and a number of funding agencies allow their grants to be used for APCs http://www.biomedcentral.com/info/about/apcfaq#grants. APCs pay for online submission and efficient peer review, for the article to be freely and universally accessible in various formats online, and for the processes required for inclusion in PubMed and archiving as described above.

### Benefits of open access

Open access was started 10 years ago by Public Library of Science (PLoS) and separately by BioMed Central. Open access is growing in popularity as libraries fill up with paper-based journals. By a recent report, up to 11% of all peer-reviewed journals and about 20% of peer-reviewed articles are now freely available through open access [3]. Furthermore, there is evidence that open access, either self-selected or mandated increases the citation impact for high quality research because they are freed from the constraints of obtaining access to subscriber-only journals [4]. Authors who have published review articles in particular, have been very surprised by the high number of 'hits' their articles have received. With open access, there is no constraint on article length and no extra cost for coloured illustrations or supplementary material.

### Fluids and Barriers of the CNS

We are launching the new look journal with a newly-designed website and some key articles covering the following topics: historical aspects of the subject, a review on comparative molecular aspects of the blood-CSF and blood-brain barriers, reviews on barrier mechanisms for immune surveillance, on intracranial pulsatility, on microvascular pericytes and on cancer cells in CSF, a commentary on drug transport via the CSF, and some key research articles. Similar to *Cerebrospinal Fluid Research*, we aim only to publish good science and will strive to maintain a high standard and fast route for publication. **We invite you to submit your articles **through the online system http://www.fluidsbarrierscns.com/manuscript/ and help us to make this a top class journal serving all researchers with an interest in brain barriers and fluids.
